# Role of RND Efflux Pumps in Drug Resistance of Cystic Fibrosis Pathogens

**DOI:** 10.3390/antibiotics10070863

**Published:** 2021-07-15

**Authors:** Viola Camilla Scoffone, Gabriele Trespidi, Giulia Barbieri, Samuele Irudal, Elena Perrin, Silvia Buroni

**Affiliations:** 1 Department of Biology and Biotechnology “Lazzaro Spallanzani”, University of Pavia, 27100 Pavia, Italy; viola.scoffone@unipv.it (V.C.S.); gabriele.trespidi01@universitadipavia.it (G.T.); giulia.barbieri@unipv.it (G.B.); samuele.irudal@iusspavia.it (S.I.); 2 Department of Biology, University of Florence, 50019 Sesto Fiorentino, Italy

**Keywords:** RND efflux pumps, drug resistance, cystic fibrosis

## Abstract

Drug resistance represents a great concern among people with cystic fibrosis (CF), due to the recurrent and prolonged antibiotic therapy they should often undergo. Among Multi Drug Resistance (MDR) determinants, Resistance-Nodulation-cell Division (RND) efflux pumps have been reported as the main contributors, due to their ability to extrude a wide variety of molecules out of the bacterial cell. In this review, we summarize the principal RND efflux pump families described in CF pathogens, focusing on the main Gram-negative bacterial species (*Pseudomonas aeruginosa*, *Burkholderia cenocepacia*, *Achromobacter xylosoxidans*, *Stenotrophomonas maltophilia*) for which a predominant role of RND pumps has been associated to MDR phenotypes.

## 1. Introduction

According to the Cystic Fibrosis Foundation Patient Registry, worldwide more than 70,000 people suffer from Cystic Fibrosis (CF) [1]. Mutations in the Cystic Fibrosis Transmembrane conductance Regulator (CFTR) gene are responsible for the insurgence of a pathological condition, with different severities based on the type of mutation [2]. The CFTR channel is required for the homeostatic control of chloride and bicarbonate ions in the lung. Its malfunctioning leads to mucin overproduction along airways and disruption of the regular mucociliary activity [3,4]. Together, these defects promote polymicrobial proliferation in the respiratory tract, where bacteria are trapped in the mucus and their clearance becomes harder and harder [4]. Moreover, their presence stimulates an exaggerated inflammatory response, making CF pathology characterized by a progressive loss of lung function.

It is noteworthy that the microbial community in CF lungs changes during the lifetime: in 3–5 year-old children, one or a few CF pathogens are detected [1], while in adolescents and adults a polymicrobial community or the prevalence of one typical CF bacterium (e.g., *Pseudomonas*, *Staphylococcus*, *Stenotrophomonas*, or *Burkholderia*) has been reported [5]. The introduction of CFTR modulator therapy has greatly improved the general health conditions of CF people; however, the effects of lumacaftor-ivacaftor, tezacaftor-ivacaftor, and elexacaftor-tezacaftor-ivacaftor therapy in patients with diverse genetic backgrounds, as well as their effects on the airway microbiota, need to be addressed [6].

A major concern regards the Multi Drug Resistance (MDR) phenotype of CF lung-associated pathogens. Beside the classical drug resistance mechanisms (i.e., drug modification and inactivation, decreased membrane permeability, modification of antibiotic targets, target protection, drug efflux), during the progression of infection, *Pseudomonas aeruginosa* may switch to the mucoid phenotype, which is very difficult to eradicate [7]. In addition, the highly resistant small-colony variant phenotype of *Staphylococcus aureus* and *P. aeruginosa* may be induced by repetitive antibiotic therapy [8,9]. Also, the proportion of methicillin-resistant *S. aureus* (MRSA), together with metallo-β-lactamase-producing *P. aeruginosa* strains is worrisome [10,11]. Moreover, during the COVID-19 pandemic, the increased usage of antibiotics to control secondary bacterial infections may further accelerate the spread of antibiotic resistance among nosocomial pathogens [12].

Indeed, while early infections by CF pathogens can be intermittent and involve different strains with multiple levels of antibiotic resistance (AR) profiles, subsequently, people with CF are chronically colonized with well adapted strains with properties (among which high levels of MDR) that differ significantly from those exhibited by the isolate which gave rise to the infection [13,14].This change is related to the adaptation of bacteria to the fluctuating and heterogeneous conditions of the CF lung environment, which exerts a high selective pressure [15]. CF lung is indeed an ecological niche characterized by several selective elements, including the host immune response, the oxidative stresses especially derived from the liberation of reactive oxygen species (ROS) by neutrophils, the interactions among different microorganisms, the nutrient availability, the modified acidity and salinity of the surrounding environment, and the oxygen deprivation in mucus [14,15,16]. Moreover, a strong selective pressure is exerted by the high levels of antibiotics used to treat the infections caused by CF pathogens (a summary of the antibiotic treatment used for the CF pathogens described in this review is reported in Supplementary Table S1) [14,15,16].

Among the consequences of this high selective pressure, there is the emergence of hypermutable strains, whose presence has been strongly associated with bacteria adaptation to the lung environment [12,13]. Hypermutable strains, together with the characteristic transition from the planktonic to the biofilm lifestyle of CF pathogens during chronic infections, lead to the development of high levels of AR in strains adapted to the CF lung. Together, all these factors increase the rate of AR through horizontal gene transfer [12,13]. Although no single mutations can lead to MDR profiles, the use of all antibiotics is prone to be compromised by the acquisition of mutations that can lead to overexpression of efflux pumps, hyperproduction of antibiotic degrading enzymes, porin loss or altered antibiotic targets [13]. Among efflux pumps, those belonging to the Resistance-Nodulation-cell Division (RND) family are able to translocate different molecules (including drugs) out of the bacterial cell in an aspecific manner, thus increasing the ability of bacteria to resist a wide range of treatments [17] RND efflux systems are tripartite complexes composed of an inner membrane protein, a periplasm associated subunit (membrane fusion protein or MFP), and an outer membrane protein (OMP), that span the inner and outer Gram-negative membranes. These pumps are activated by a proton motive force to export compounds into the extracellular environment. The best-described members of this family are the AcrAB-TolC and the MexAB-OprM of *Escherichia coli* and *P. aeruginosa*, respectively [18,19].

In this review, we will describe the principal RND efflux pump families which have been found in CF pathogens, then we will focus on the main Gram-negative bacterial species (*P. aeruginosa*, *Burkholderia cenocepacia*, *Achromobacter xylosoxidans*, *Stenotrophomonas maltophilia*) for which a predominant role of RND pumps has been associated to MDR phenotypes. 

## 2. RND Efflux Pump Families in CF Pathogens

The RND superfamily is a ubiquitous group of efflux pumps conserved in all domains of life (for a recent review see [17]). This superfamily is divided into nine functionally recognized families, six of which have representatives in Gram-negative bacteria [17,20].

In particular, the SecDF efflux pumps are involved in the general secretion (Sec) pathway and members of this family are present in both Bacteria and Archea [17,20]. However, most of the characterized RND proteins of Gram-negative bacteria belong to the Hydrophobe/Amphiphile Efflux 1 (HAE-1) and Heavy Metal Efflux (HME) families, involved in the export of multiple drugs and heavy metals respectively [17,20]. In addition, three other families with few representatives have been found in Gram-negative bacteria that are less known and characterized: (i) the Nodulation Factor Exporter (NFE) family that was identified as a probable nodulation factor exporter, although recently added members of this family are drug exporters; (ii) the Aryl Polyene Pigment Exporters (APPEs), that have been found in *Xanthomonas oryzae* where they are involved in exporting a pigment [17,20]; (iii) the Hydrophobe/Amphiphile Efflux 3 (HAE-3) family that included some Archea transporters but also HpnN proteins, a group of Gram-negative pumps apparently involved in the transport of hopanoids to the outer membrane [17,20]. The RND proteins of the HAE-1, HME and NFE families are generally associated with an MFP and an OMP protein to form a complex that allows the extrusion of substrates directly out of the cells. The genes coding for these three proteins are usually associated in an operon [17,20].

Most of the RND systems identified and experimentally characterized in cystic fibrosis pathogens belong to the HAE-1 family and are involved in antibiotic efflux. In *P. aeruginosa*, twelve different RND operons have been found (*mexAB*-*oprM*, *mexCD*-*oprJ*, *mexEF*-*oprN*, *mexXY*, *mexJK*, *mexGHI*-*opmD*, *mexPQ*-*opmE*, *mexMN*, *muxABC*-*ompB*, *mexVW*, *triABC* and *czcABC*) [21]. The CzcABC system belongs to the HME family, while all the others belong to the HAE-1 family [22]. All the twelve systems have been experimentally characterized and most of them are conserved among different strains (in particular, MexAB-OprM, MexCD-OprJ, MexEF-OprN, MexXY and MexJK) [22,23,24,25,26].

In the *Burkholderia cepacia* complex at least 19 different putative HAE-1 RND efflux pumps are present, four of which (operon RND-4 or *bpeAB*-*oprB*, operon RND-6 RND-7, operon RND-10 or *ceoAB*-*opcM* or *bpeEF*-*oprC* and operon RND-13) are being conserved among several different strains [20,27,28]. Most of these proteins belong to the HAE-1 family and for several of them, the role in antibiotic efflux have been experimentally confirmed in several *Burkholderia* species (RND-1, RND-3 or AmrAB-OprA, RND-4 or BpeAB-OprB, RND-8 and RND-9, RND-10 or CeoAB-OpcM or BpeEF-OprC) [29]. Moreover, for two systems (RND-11 or CusABC and RND-12 or CzcABC) identified as belonging to the HME family [20,30], the role in heavy-metal efflux has been experimentally validated [31]. The genes coding for putative SecDF, HpnN/HAE-3 and APPE proteins have been found but not experimentally confirmed [20]. Finally, in this genus, a group of operons which appear not to belong to any of the recognized RND families have been identified and defined as Uncertain Function (UF) [20,30]. 

In the genome of the type strain of *A. xylosoxidans*, ATCC 27061, the genes coding for 9 different RND efflux pumps have been identified [32]. Three of these efflux pumps have been functionally characterized: AxyABM (homolog of MexAB-OprM) [33], AxyXY-OprZ (with homology to MexXY-OprM) [34] and AxyEF-OprN [35]. All these systems are involved in the transport of several different antibiotics and belong to the HAE1 family of RND transporters [33,34,35]. The substrates and the family of the other six pumps have yet to be determined. A comparative genomic analysis showed that the genes coding for most of these nine systems are conserved among different *A. xylosoxidans* strains [34], with one of them, *axyABM*, conserved in all the sequenced *Achromobacter* genomes [36], while *axyXY–oprZ* has been found also in *Achromobacter ruhlandii* [37]. Regarding proteins belonging to the HME family, RND transport systems homologous of CzcABC and CusABC are present in the genomes of other *Achromobacter* strains [38,39].

Finally, in *S. maltophilia*, the genes coding for fifteen putative HAE-1 RND systems have been found, seven of which (*smeVWX*, *smeYZ*, *smeGH*, *smeMN*, *smeOP*, *smeDEF*, *smeIJK*) seem to be conserved among different strains [40,41]. Eight out of these fifteen pumps (SmeVWX, SmeYZ, SmeOP, SmeDEF, SmeIJK, SmeABC, SmeGH, SmeMN) have also been experimentally characterized, confirming that they are actually involved in AR [40]. In addition, the genes coding for six others putative HME RND efflux pumps have been found in the genome of the K279a strain [41], but none of them have been experimentally validated.

## 3. RND in *Pseudomonas aeruginosa*

### 3.1. Pseudomonas aeruginosa Infections in CF

*P. aeruginosa* is a Gram-negative bacterium that belongs to the family of *Pseudomonadaceae*. Thanks to its metabolic versatility it is able to colonize many different environments and to establish opportunistic infections [42]. The World Health Organization classified as a priority one *P. aeruginosa* carbapenem resistant [43]. *P. aeruginosa* is the most common causative agent of Gram-negative nosocomial infections and lung infection in CF patients [44]. MDR *P. aeruginosa* is responsible for over 72,000 infections and 4800 deaths annually in Europe and the majority of these cases are attributed to carbapenem and colistin-resistant strains [45].

*P. aeruginosa* has a relatively large genome of 5.5–7 million base pairs, encoding a large number of regulatory enzymes involved in metabolism, transport and efflux [46]. During childhood, CF patients are colonized by both *P. aeruginosa* and *S. aureus*, while in adulthood *P. aeruginosa* is predominant and induces lung function decline [47]. The interaction between *P. aeruginosa* and its hosts is still poorly understood and its persistence in the airways is due to highly complex and multifactorial reasons [48]. The CF airways environment helps *P. aeruginosa* colonization over other bacteria (*S. aureus*) and the consequence of this is the prevalence of *P. aeruginosa* in adults, ranging from 31 to 47% [49]. One possible reason for this prevalence is that the physiological defects linked to CFTR mutations (such as mucus viscosity, production of reactive oxygen species, impaired autophagy, reduced airway acidity and accumulation of ceramides) induce advantages to *P. aeruginosa* [50].

During the course of the infection, the genetic and phenotypic traits of *P. aeruginosa* strains in CF airways are subjected to evolutionary changes in response to the selective pressure of the environment [51]. Chronic *P. aeruginosa* infections are recalcitrant to antibiotic treatment, which are extremely challenging due to the ability of the bacterium to resist the commonly used compounds thanks to its numerous mechanisms of resistance (efflux pumps, ability to form biofilm, persistence) [52]

*P. aeruginosa* is resistant to numerous antibiotics belonging to the aminoglycosides, quinolones, and β-lactams families [53]. Mechanisms of AR of *P. aeruginosa* are classified into intrinsic, acquired, and adaptive. Mechanisms of intrinsic AR are encoded by the core genome of the organism, adaptive resistance is induced by environmental stimuli, while acquired resistance depends on the gain of resistance genes derived from other organisms or those which originated after the selection of mutations [54]. Among intrinsic resistance mechanisms there are: the low outer membrane permeability, the expression of efflux pumps, lipopolysaccharides modification, and the production of enzymes that inactivate antibiotics. The adaptive resistance is related to biofilm formation that limits antibiotic access to bacterial cells, decreases bacterial motility and promotes the formation of persister cells [55]. Acquired resistance is the result of horizontal transfer of resistance-related genes or of mutational changes [56].

### 3.2. Pseudomonas aeruginosa RND Efflux Systems

Antibiotic extrusion and resistance in *P. aeruginosa* can be closely related to tripartite RND efflux pumps [57]. Efflux pumps are also involved in cellular stress response. Stress signals such as host factors, detergents and endogenous inducers of bacterial stress could help to select mutants, which over-express efflux systems [58]. The constant inflammation of CF lungs exposes *P. aeruginosa* to reactive oxygen species (ROS), which might induce the prevalence of strains over-expressing efflux pumps (MexAB-OprM and MexXY-OprM) [59]. Moreover, Fraud and colleagues showed that ROS over-exposure selects resistant mutants expressing the RND MexXY-OprM [60].

Among the 12 RND efflux pumps identified in *P. aeruginosa,* six contribute to AR [61]. These RNDs are: MexAB-OprM, MexCD-OprJ, MexEF-OprN, MexXY-OprM, MexJK-OprM and MexVW-OprM (Table 1) [52,62]. MexAB-OprM and MexXY-OprM are constitutively expressed at the basal level in wild type strains and are induced by antibiotic substrates, while the other systems are not expressed in wild type strains [52,63]. The genes encoding these tripartite efflux pumps are organized in operons, but in certain cases the operon does not contain the OMF gene, such as in the case of MexXY, MexJK and MexVW.

MexAB-OprM extrudes carbapenems, chloramphenicol, fluoroquinolones, lincomycin, macrolides, novobiocin, tetracyclines, and all β-lactams except imipenem. It is also involved in the efflux of triclosan (antiseptic compound) and of sodium dodecyl sulfate (surfactant). While deletion of *mexAB-oprM* results in a *P. aeruginosa* strains sensitive to all the above-mentioned antibiotics, a mutant overexpressing MexAB-OprM is characterized by a significant level of resistance [64,65]. The efflux pump MexAB-OprM is composed of an inner membrane protein MexA, a fusion protein MexB and the outer membrane protein OprM [66]. Genes encoding these proteins constitute an operon which is controlled by the transcriptional regulator MexR [67]. The *mexR* gene is localized upstream of the *mexAB-oprM* operon and encodes a transcriptional repressor which binds the intergenic region between *mexA* and *mexR*, in proximity to their promoters [68]. When MexR is not functional, there is MexAB-OprM overexpression. *P. aeruginosa* clinical isolates showed different types of *mexR* mutations, leading to the production of a protein unable to dimerize, to bind the DNA and to repress *mexAB-oprM* operon or mutations that result in the complete absence of a functional MexR (such as peptide premature termination) [69,70]. A recent study focused on the evolution of resistance during infections showed that the frequency of mutations (frameshift in either *mexA* or *oprM*) in *mexAB-oprM* rises rapidly during infection, providing evidence that the loss of this pump is adaptive [71]. Mutants have a low meropenem resistance, suggesting that these mutations arise in a sub-population of cells of the ancestral strain that are protected from meropenem by physical barriers, such as biofilm, or by phenotypic resistance (tolerance or persistence) [72,73].

The RND efflux pump MexCD-OprJ is expressed in *nfxB P. aeruginosa* mutants only. NfxB is the negative regulator of MexCD-OprJ and clinical isolates with diverse mutations in *nfxB* gene were isolated. These mutants showed different levels of resistance to the antibiotics effluxed by MexCD-OprJ, such as chloramphenicol, erythromycin, fluoroquinolones, and tetracyclines [74]. 

Another RND efflux pump is MexEF-OprN that, unlike the other efflux systems, is positively regulated by the transcriptional activator MexT [64]. This efflux pump extrudes chloramphenicol, fluoroquinolones, tetracycline, and trimethoprim [75]. In most laboratory strains deriving from reference *P. aeruginosa* strain PAO1, the *mexT* gene is frequently unfunctional, causing the suppression of *mexEF-oprN* operon [76]. On the other hand, when MexT is active, it also works as a repressor of the OprD porin, inducing an increase of resistance to carbapenem [77]. *P. aeruginosa* mutants in the *nfxC* gene (norfloxacin resistance gene) are characterized by the over-expression of *mexEF-oprN* operon and are more resistant to chloramphenicol, fluoroquinolones, tetracycline, trimethoprim, and imipenem [78]. 

One of the most studied RND of *P. aeruginosa* is the efflux system MexXY-OprM, which contributes to intrinsic resistance to aminoglycosides, tetracyclines, erythromycin, and cefepime [79]. The MexXY can form functional complexes with two different outer membrane proteins, OprM and OprA, in *P. aeruginosa* PA7 [80]. Recently, it has been shown that the substrate specificities of MexXY can change depending on which OM protein it complexes with [81]. Both OprM and OprA are involved in aminoglycosides efflux, while carbenicillin and sulbenicillin are substrates only of the MexXY-OprA complex [81]. The regulator of this RND is the repressor MexZ and mutations in its gene, or in the regulatory region, lead to overexpression of MexXY [82,83]. In *P. aeruginosa* CF clinical isolates, the most common mutations are localized in the *mexZ* gene, inducing MexXY-OprM overproduction. These *mexZ* mutations arise during chronic infections in CF patients, contributing to tobramycin resistance, one of the first-line antibiotics used in CF [84]. The expression of *mexY* and *mexZ* was found to be higher in adults with chronic infection than in children with new or chronic infections, suggesting that these mutations are subjected to positive selection [85].

Another RND efflux pump, MexJK, was identified using triclosan (biocide) as selective agent in *mexL* mutants in a Δ*mexAB-oprM* and Δ*mexCD-oprJ* strains [86]. Furthermore, MexCD-OprJ expression is selected by triclosan and could be considered an interesting selective tool to study efflux systems [86] MexJK expression is controlled by the product of an upstream regulatory gene, *mexL*, similar to what has been described in other RND efflux pumps. MexJK lacks its own outer membrane protein and requires OprM for the efflux of antibiotics [86].

Using a *P. aeruginosa* mutant lacking *mexAB*, *mexCD*–*oprJ*, *mexEF*–*oprN* and *mexXY*, the RND efflux pump MexVW was characterized [87]. In the proximity of the *mexVW* genes, no ORFs are present that could encode a regulatory protein; similarly, no genes coding for an outer membrane protein are present in the downstream region. MexVW works as a multidrug efflux pump and uses OprM as OMP. Overexpression of *mexVW* was demonstrated to confer resistance to norfloxacin, ofloxacin, chloramphenicol, cefpirome, tetracycline, and ethidium bromide [87].

### 3.3. P. aeruginosa RND Efflux Pumps Inhibitors

Among the *P. aeruginosa* efflux pump inhibitors, the most studied is Phe-Arg-β- naphthylamide (PAβN), a broad spectrum peptidomimetic compound. PAβN was shown to interfere with the four RND systems of *P. aeruginosa*: MexAB-OprM, MexCD-OprJ, MexEF-OprN, MexXY-OprM. The association of chloramphenicol, fluoroquinolones, macrolides, ketolides, oxazolidinones, and rifampicin with PAβN increases their effects [88]. PAβN functions as substrate of Mex efflux pumps and competes with antibiotics, preventing their extrusion [89]. Unfortunately, PAβN and its derivatives during phase 1 clinical trials showed adverse toxicity and pharmacokinetic profile [90].

Another efflux pump inhibitor is the pyridopyrimidine derivative D13-9001 [89]. It blocks MexAB-OprM in vivo and in vitro and it showed low toxicity profiles [91]. The mechanism of action of this compound relies on a tight interaction with the hydrophobic trap of the pump, preventing its conformational changes. At the same time, D13-9001 blocks the substrate binding to MexB [92]. The limit of this molecule is its specificity for MexAB-OprM: in fact, efflux pump inhibitors should be broad spectrum compounds in order to be used as adjuvants together with antibiotics that are substrates of several efflux pumps. Moreover, different mechanisms of resistance were identified when the compound was administered with carbenicillin. The resistance occurred due to a mutation in the residue F628 of MexB, a site involved in inhibitor binding [93,94].

A polyamine scaffold was identified as an efflux pump inhibitor by Fleeman and co-workers [95]. Polyamines are essential organic polycations ubiquitous in all forms of life and are composed of an aliphatic carbon chain with numerous amino groups. Five polyamine derivatives were demonstrated to potentiate the effect of aztreonam, chloramphenicol, and tetracycline, inducing an MIC_90_ decrease of 5- to 8-fold. These compounds have limited toxicity and no inhibitory effects on the eukaryotic Ca^2+^ channel of human kidney cells [95].

Among the natural products that target MDR efflux pumps, there are EA-371α and EA-371δ, identified by screening a library of 78,000 microbial fermentation extracts [96]. These compounds are the products of a *Streptomyces* strain and are potent MexAB-OprM inhibitors, with a MPC_8_ (minimum potentiation concentration decreasing the MIC of 8-fold) values of 4.29 μM (EA-371α) and 2.15 μM (EA-371δ) for levofloxacin against strain PAM103. Unfortunately, EA-371α could not be considered a lead compound because of its moderate cytotoxicity [96].

Another type of RND efflux inhibition relies on the application of phage therapy. While the traditional phage therapy is based on the administration of phages to block bacterial cell growth, another approach used phages to steer AR evolution. An example is the lytic *Myoviridae* bacteriophage OMKO1 that uses OprM as a receptor binding site. Bacteria resistant to OMKO1, lacking OprM, are more sensitive to ciprofloxacin, tetracycline, ceftazidime, and erythromycin due to the counterselection of MDR *P. aeruginosa* and, possibly, to a change in the efflux pump mechanism [97].

## 4. RND in *Burkholderia cenocepacia*

### 4.1. Burkholderia cenocepacia Infections in CF

*Burkholderia cepacia* complex (Bcc) species are abundant in the polymicrobial communities inhabiting the lungs of adult CF patients [98]. Within this group of 24 phenotypically related but genetically distinct bacterial species, *Burkholderia cenocepacia* and *Burkholderia multivorans* are responsible for approximately 70–85% of all Bcc infections in this cohort of patients [99,100]. The wide variety of potential virulence factors (e.g., catalases, proteases and siderophores) produced by these bacteria to evade host defenses, their innate resistance to many antibiotics and disinfectants, their ability to adhere and invade epithelial cells and to survive inside macrophages, render *B. cenocepacia* infections very difficult to treat [101,102,103,104]. Clinical effects vary from transient carriage to chronic lung infection, which can rapidly deteriorate to necrotizing pneumonia and sepsis, the so-called “cepacia syndrome”, resulting in a significant decrease in patients’ survival [105,106]. Moreover, the poor post lung transplant outcomes of individuals affected by *B. cenocepacia* renders chronic infection as a contraindication for lung transplantation [107]. In this scenario, despite the relatively low and stable prevalence of *B. cenocepacia* infections, affecting around 3% of CF patients in Europe [108], this opportunistic pathogen represents a serious burden for the management of people affected by CF.

The main challenges in the treatment of *B. cenocepacia* infections are represented by the intrinsic resistance of this species to clinically relevant antibiotics and by their tolerance to antibiotic exposure, typically associated with a biofilm lifestyle [109,110]. In the absence of evidence-based guidelines for treatment [111], various therapeutic protocols based on the use of single or multiple antibiotics administered by different routes (intravenous, oral, inhaled, or combined) for varying periods of time have been employed in clinics. However, complete eradication of the infection is difficult to achieve [112,113]. Strategies based on compounds that improve the activity of antibiotics (helper compounds) by blocking the main resistance mechanisms or altering the physiological state of antibiotic-tolerant cells are in clinical trials or under study [114,115,116,117]. These molecules generally act by impairing bacterial growth, permeabilizing bacteria through the alteration of the structure of the outer membrane, inhibiting biofilm formation and eradicating established biofilms [114,116]. Alternative approaches based on molecules used for other diseases, natural products, quorum-sensing inhibitors and antimicrobial peptides are under investigation [118,119,120,121]. Finally, interest in the design of *B. cenocepacia* vaccines has recently risen [122].

### 4.2. Burkholderia cenocepacia RND Efflux Systems

The ability to produce a variety of efflux pumps significantly contributes to the inherent multidrug resistance of *B. cenocepacia* [112,123]. After the identification of the gene cluster encoding the conserved salicylate-regulated RND-10 efflux pump responsible for chloramphenicol, trimethoprim, and ciprofloxacin resistance [124], sixteen genes encoding transporters of the RND family, organized in 14 operons, have been identified in the genome of the reference *B. cenocepacia* strain J2315 (Table 2) [20,125,126]. This CF isolate, belonging to the highly transmissible epidemic ET12 lineage, was used for the preparation of a collection of mutant strains, each carrying a marker-less deletion of a single RND operon, thus allowing the investigation of the role of these systems in *B. cenocepacia* physiology and antibiotic susceptibility [127,128,129]. While the RND-deleted strains did not show any defect in their growth characteristics, the absence of a few specific RND-systems resulted in increased antibiotic susceptibility and, in some cases, alterations in the production of biofilm matrix compared to their parental strain [129]. In particular, when grown in planktonic cultures, mutants lacking the RND-3 and RND-4 efflux systems displayed a higher susceptibility to both ciprofloxacin and tobramycin and a reduced secretion of quorum-sensing molecules [127,129]. Interestingly, lifestyle specific effects could be observed for the different mutants. While the contribution of the RND-3 system to the intrinsic AR of *B. cenocepacia* J2315 was exerted both in planktonic and sessile cells, the RND-4 efflux pump played a major role in the efflux of ciprofloxacin, tobramycin, minocycline, and chloramphenicol only in planktonic cells. On the contrary, the RND-8 and RND-9 efflux systems were demonstrated to confer protection against tobramycin only in biofilms, but not in planktonic cultures [129].

The lifestyle-specific activity of these pumps appears as a cellular response to regulatory signals governing the physiology of the cell. In fact, besides contributing to the extrusion of antibiotics and of a variety of compounds toxic for cellular metabolism, RND systems play a role in the control of physiological processes and virulence of *B. cenocepacia* [128]. Deletion of RND-efflux pumps was reported to affect motility-related phenotypes and biofilm formation, with RND-4 and RND-9 mutant deletion strains showing an enhanced biofilm formation ability and an increased and reduced swimming motility, respectively [128]. As revealed by transcriptomic analysis, while the motility phenotype could be easily correlated to a differential expression of motility genes in the mutant strains compared to wild type, the increased ability to form a biofilm could not be linked to an altered expression of genes involved in biofilm formation, suggesting indirect regulatory mechanisms, possibly activated by altered concentrations of toxic compounds or metabolic signals that accumulate in the cell as a consequence of efflux pump inactivation.

The presence of multiple operons encoding RND-efflux pumps in the *B. cenocepacia* genome suggests a functional redundancy and synergistic activity, accounting for the lack of alterations in the phenotype and in the antibiotic susceptibility of the majority of single RND deletion mutants [127,129]. Interestingly, the high level of conservation of the RND-4 operon in the genomes of *Burkholderia* species is consistent with the multiple functions in which this system is involved and with the effects of its inactivation on the increased susceptibility to different antimicrobial compounds, including essential oils and disinfectants [127,130,131,132]. On the other hand, RNDs with a narrow phylogenetic distribution, like RND-9, show a more specific activity, with consequent milder phenotypic changes observed in the corresponding J2315 deletion strain [128,129]. Noteworthy, when the conserved RND-4 efflux pump is missing or inactivated, overexpression of the RND-9 system can compensate for its function. For example, in a *B. cenocepacia* RND-4 deletion strain, mutations in a gene (*bcam1948*) encoding a transcriptional repressor of the RND-9 operon were demonstrated to confer resistance to a new antitubercular thiopyridine compound whose antimicrobial activity was previously demonstrated to be impaired by RND-4 mediated extrusion [130,133]. Interestingly, mutations in the same regulator confer resistance to a 2,1,3-benzothiadiazol-5-yl family compound and to multiple antibiotics (chloramphenicol, ciprofloxacin, levofloxacin, norfloxacin, sparfloxacin and nalidixic acid) [134]. It is noteworthy that, despite the important contribution of RND-4 in facilitating multiple AR in the *B. cenocepacia* J2315 laboratory strain, no significant differences in the expression of the *RND-4* gene (*bcal2822*) was detected in multidrug-resistant clinical isolates which, on the contrary, displayed a high expression level of *RND-3* (*bcal1674*) and *RND-9* (*bcal1947*) [135]. However, the upregulation of the genes encoding RND-6 and RND-4 were found to be involved in conferring resistance to different classes of antimicrobials (aminoglycosides, *β-*lactams, fluoroquinolones, folate-pathway inhibitors) in a clonal variant of *B. cenocepacia* isolated during long-term infection in CF lungs [136].

Phylogenetic analysis revealed a high degree of sequence similarity between *RND-4* and the functionally distinct *RND-2* operon, encoding a system present in only some Bcc species [27]. RND-2 is not expressed in bacteria growing in LB medium and its ability to confer resistance to fluoroquinolones, tetraphenylphosphonium, streptomycin and ethidium bromide could be identified only by overexpression experiments in *E. coli* [125]. Noteworthy, RND-2 overexpression is able to restore resistance to some antibiotics in an RND-4 deletion mutant, supporting the hypothesis that this operon originated from an RND-4 duplication event that led to the creation of a system maintaining the ancestral substrate specificity but subjected it to different regulatory mechanisms [28].

## 5. RND in *Achromobacter xylosoxidans*

### 5.1. Achromobacter Infections in CF

The *Achromobacter* genus consists of 19 species [137] of motile, non-lactose fermenting Gram-negative environmental bacilli isolated from soil and water sources. Even though they are not intrinsically pathogenic bacteria, they can represent a threat for critically ill, immunocompromised and CF patients. *A. xylosoxidans* has been known to cause pulmonary infections in CF patients since the 1980s [138], but only recently has it been recognized as one of the main CF pathogens. There is a high regional variability in its infection rate [139] but different reports highlight a worldwide rise in prevalence [140,141,142]. This increase could be due both to the selective antimicrobial pressure present on the CF lung bacterial community, and to the recent improvement of the detection methods, which allow the unequivocal identification of *Achromobacter* isolates at the species level [143]. This highlighted the presence in CF of different species aside from *A. xylosoxidans*, which still remains the most prevalent, such as *Achromobacter ruhlandii*, *Achromobacter dolens*, and *Achromobacter insuavis* [143]. Although the impact of these infections on lung function is not fully understood yet [144,145], it is known that these bacterial species, so closely related to the pathogenic *Bordetella* genus, have a high host adaptation potential, possessing several virulence-associated genes [146].

The treatment of *Achromobacter* spp. infections is extremely challenging since they show inherent resistance to most penicillins and cephalosporins, as well as to aztreonam, fluoroquinolones, and aminoglycosides [147]. Besides the intrinsic resistance mechanisms, *Achromobacter* often exhibits acquired resistances, especially towards β-lactams, but also to aminoglycosides and trimethoprim, achieved by horizontal gene transfer [148]. This array of resistance determinants makes these bacteria potentially resistant to every class of antibiotics, and cases of pan-drug-resistant *Achromobacter* spp. have been already reported [149]. For this reason, the optimal antibiotic therapy for these infections is patient-specific, even if piperacillin–tazobactam, trimethoprim–sulfamethoxazole, and meropenem are usually the most active agents [147]. Concerning the innate resistance mechanisms, initially some β-lactamases were biochemically characterized [150,151,152,153], but the class D β-lactamases OXA-114, in *A. xylosoxidans*, and OXA-258, in *A. ruhlandii*, are nowadays the best characterized enzymes, although their role in the β-lactams resistance profile is likely secondary [37,154]. To better study the resistance potential of *A. xylosoxidans*, Hu and colleagues performed a genome-wide analysis, predicting the presence of 50 drug resistance genes, 38 of which were efflux pump genes [32].

### 5.2. Achromobacter spp. RND Efflux Systems

The genome of *Achromobacter* spp. contains a significantly higher number of efflux pump-related genes compared with other genera [155]. Only three of nine RND efflux systems have been studied so far (Table 3), and a lot of work is still needed to have a comprehensive overview of the intrinsic and acquired antimicrobial resistance patterns in the *Achromobacter* genus.

The first RND-type multidrug efflux system described in *A. xylosoxidans* (even though the strain used in this work was later reclassified as *A. insuavis*) was the AxyABM [33]. This RND system is the ortholog of the MexAB-OprM system of *P. aeruginosa* (60–72% protein identity) and shares with it the same operon organization. Indeed, the genes composing the multiprotein complex are grouped in a cluster of three open reading frames, *axyA* (the MFP), *axyB* (the RND transporter protein), and *axyM* (*oprM*; the OMP). Moreover, upstream of the operon a gene coding for a transcriptional regulator, namely *axyR*, is present, as already seen in *P. aeruginosa* for *mexR*, although the two genes do not share any homology [33]. By inactivation of *axyB*, it was also demonstrated that the spectrum of activity of AxyABM is comparable, even if not identical, to the one of MexAB-OprM, being involved in the innate resistance to a broad spectrum of antibiotics, in particular most cephalosporins and aztreonam, but also nalidixic acid, fluoroquinolones, and chloramphenicol [33]. This RND system is present in all the sequenced *Achromobacter* genomes [146], but it was better characterized only in *A. ruhlandii*, where it seems to have a narrower spectrum of activity. Indeed, by cloning the *axyABM* operon in *E. coli*, it was demonstrated to be only involved in the extrusion of chloramphenicol, nalidixic acid and trimethoprim/sulfamethoxazole [37]. Finally, besides the innate antibiotic tolerance, AxyABM is probably involved also in persistence and biofilm metabolism of *A. xylosoxidans*, since the gene *axyA* was found to be 21-fold upregulated upon the establishment of chronic infections in CF lungs [156]. Moreover, in the same strain, the expression of *axyA* increased more than 7-fold in sessile cells, highlighting the importance of this efflux system in biofilm formation [156].

To identify the mechanism(s) responsible for the high-levels of innate resistance of *A. xylosoxidans* towards aminoglycosides, a genomic comparison with *P. aeruginosa* was performed. This approach led to the characterization of the AxyXY-OprZ efflux pump, the ortholog of the MexXY-OprM RND system of *P. aeruginosa* [34]. AxyXY-OprZ is encoded by an operon conserved in many *Achromobacter* species, predominantly in those often recovered from CF patients, and it is described as the major resistance mechanism to aminoglycosides, since its presence is always associated with a resistant phenotype, whereas its absence leads to a sensitive phenotype [157].

The *axyXY-oprZ* operon is under the negative control of AxyZ, a TetR-type transcriptional repressor homolog of the *P. aeruginosa* MexZ and is encoded by the gene *axyZ*, found upstream of the cluster [158]. Surprisingly, this transcription factor plays a role also in the regulation of a novel carbapenemase, Axc, highly expressed in meropenem-resistant *A. xylosoxidans* clinical isolates [159]. Indeed, loss of function mutations in the *axyZ* sequence, and especially the V29G substitution localized in the DNA-binding domain of the protein, lead to the overexpression of AxyXY-OprZ, but also of the Axc carbapenemase, increasing the MICs of antibiotic substrates of these proteins [158,159]. This demonstrates that AxyZ is involved in a wide regulatory pathway controlling the activation of disparate AR mechanisms. The AxyZ mutations can be quite easily selected in vitro by exposure of the bacterium to aminoglycosides [158], a class of antibiotic extensively used for CF infections treatment, and consequently these are reported to be frequently associated with the pathoadaptive process of *A. xylosoxidans*, *A. ruhlandii* and *A. insuavis* in CF lung [160].

The AxyXY-OprZ possesses the ability to extrude a broad spectrum of antibiotics, since its inactivation leads to a drastic decrease in the MICs of aminoglycosides and, to a lesser extent, of carbapenems, cefepime (the only cephalosporin not extruded by AxyABM), ceftazidime, some fluoroquinolones, tetracyclines, and erythromycin. Moreover, this RND pump seems to be partially involved in the *Achromobacter* spp. acquired resistance to carbapenems, since its impairment leads to a significant decrease of carbapenem MICs in a resistant clinical isolate [34]. However, the MIC value results higher than the carbapenem-sensitive *Achromobacter* strains, suggesting the presence of additional resistance mechanisms, such as the recently described carbapenemase Axc. Despite the high similarity between AxyXY-OprZ and its *P. aeruginosa* counterpart, the *Achromobacter* efflux pump confers up to a 32-fold higher level of resistance to aminoglycosides. It was hypothesized that this difference is probably due to the different Opr protein associated with the RND complex, since OprZ is the homolog of OprA (not OprM), the outer membrane protein coupled with MexXY in some *P. aeruginosa* genetic lineages [34].

The last RND efflux pump characterized in *Achromobacter* spp. was the AxyEF-OprN, the ortholog of the *P. aeruginosa* MexEF-OprN [35]. In contrast to the other two RND systems, this pump has a narrow spectrum of activity and was initially demonstrated to have a role in the *Achromobacter* innate resistance to few fluoroquinolones, carbapenems, and tetracyclines. Indeed, by analyzing the effect of *axyE* deletion in the AX08 clinical isolate, Nielsen and collaborators showed a decrease of the MIC of levofloxacin, making this strain susceptible to this antibiotic according to the EUCAST interpretative criterion for *Pseudomonas* spp. Moreover, a 2-fold decrease in the MIC of ertapenem, ciprofloxacin, and doxycycline was reported [35]. Surprisingly, in the same paper they also described an increase in the MICs of some β-lactams as a consequence of the pump inactivation, but this aspect was not further investigated [35]. AxyEF-OprN was also characterized as the main mechanism responsible for acquired fluoroquinolone resistance in *Achromobacter* [161]. Indeed, it was demonstrated that, different to many Gram-negative bacilli, the fluoroquinolones-resistant phenotype is not due to amino acid substitutions within the Quinolone Resistance Determining Regions (QRDRs) of the targets (DNA gyrase and topoisomerase IV), but it is mainly due to AxyEF-OprN overexpression. In particular, the overproduction of the efflux pump in *Achromobacter*-resistant clinical isolates is often caused by gain-of-function mutations of AxyT, the transcriptional activator of the *axyEF-oprN* operon, although the big difference found in fold change in strains owning the same mutation suggests an interplay between different regulatory pathways [161].

### 5.3. Achromobacter spp. RND Efflux Pumps Inhibitors

Until now, despite the prominent role of RND efflux pumps in *Achromobacter* innate and acquired AR, no specific inhibitors have been studied. The only active compound present in the literature is berberine, a benzylisoquinoline alkaloid isolated from many medicinal plants, and its derivatives, characterized as specific inhibitors of the *P. aeruginosa* MexXY system, but tested also against *A. xylosoxidans* [162,163]. Indeed, in this bacterium berberine significantly reduced the tolerance to aminoglycosides, decreasing the MICs of amikacin, arbekacin, gentamicin, and tobramycin (the substrates of the AxyXY-OprZ efflux pump) up to 32-fold [162]. Moreover, among eleven berberine derivatives, the 13-(2-methylbenzyl) berberine (13-o-MBB) showed the best activity against *P. aeruginosa* and thus it was tested against *A. xylosoxidans*. The presence of 13-o-MBB resulted in a further increased sensitivity to aminoglycosides, and the most impressive result was obtained in combination with gentamicin, reducing its MIC of more than 512-fold [163]. However, even low concentrations (30 μg/mL) of this molecule are cytotoxic to human cells in vitro [163], making the development of less toxic derivatives fundamental for future application in humans.

## 6. RND in *Stenotrophomonas maltophilia*

### 6.1. Stenotrophomonas maltophilia Infections in CF

*Stenotrophomonas maltophilia* is a Gram-negative, aerobic, non-fermentative bacillus, belonging to the class of gammaproteobacteria. It is an ubiquitous contaminant in soil, water, food, and hospital settings [164]. Its major presence in healthcare centers, after *Acinetobacter* spp. and *Pseudomonas aeruginosa*, is linked to opportunistic infections with relevant morbidity among patients with underlying pathologies, such as cystic fibrosis, or immunocompromised subjects, with an incidence in USA intensive care units of 4.3% of all Gram-negative infections [41,165]. Risk factors include malignancy, chronic respiratory diseases, and long-term hospitalization. In CF patients, *S. maltophilia* isolation in the respiratory tract is linked to intravenous antibiotic use and oral quinolone administration, as for the use of anti-pseudomonal antibiotics; approximately 11% of CF patients are colonized by this bacterium, even if its role in such condition is not clear [164]. *S. maltophilia* chronic infection is correlated to a lower mean percent predicted Forced Expiratory Volume in the 1st second (FEV1) compared to the uninfected control, with a significantly higher risk of pulmonary exacerbation [164]. Combinatorial treatments are efficient in avoiding clone selection, e.g., with trimethoprim-sulfamethoxazole and ticarcillin-clavulanate, doxycycline and ticarcillin-clavulanate, trimethoprim-sulfamethoxazole and piperacillin-tazobactam, ciprofloxacin and ticarcillin-clavulanate. Nevertheless, MDR strains were isolated from topical antiseptic, hand-washing soap, bottled water, and intravenous cannulae, nebulizers and prosthetic devices, showing how hazardous direct-contact transmission and how tolerant this pathogen can be [164]. Such persistence in the environment is adjuvanted by a broad array of intrinsic AR determinants against β-lactams, macrolides, aminoglycosides, cephalosporins, polymyxins, tetracyclines, chloramphenicol, fluoroquinolones, carbapenems, and trimethoprim-sulfamethoxazole [164]. Such phenotype results from the interaction of different layers, as poor membrane permeability, the presence of chromosomally encoded L1 and L2 β-lactamases [166], AAC(6′)-Iz and APH(3′)-IIc aminoglycoside-modifying enzymes [167], and multidrug resistance efflux pumps [164].

### 6.2. Stenotrophomonas RND Efflux Systems

In the *S. maltophilia* K279a strain genome eight pumps have been annotated, while seven (*smeABC*, *smeDEF*, *smeGH*, *smeIJK*, *smeOP*, *smeU1VWU2X*, *smeYZ*) of them have been characterized as hydrophobic and amphiphilic efflux (HAE)-RND pumps (Table 4) [167,168,169,170,171,172,173].

One of the first identified HAE-RND pumps has been SmeABC, which shows similarities to different efflux pumps, such as MexAB-OprM in *P. aeruginosa*, TtgABC in *P. putida* and AcrAB in *E. coli* [172]. This tripartite efflux pump, whose operon is controlled by the SmeSR sensor proteins, confers resistance to third-generation β-lactams, aminoglycosides, and fluoroquinolones and leads to trimethoprim susceptibility once overexpressed [166,172], while physiologically it does not confer intrinsic resistance due to its low-basal expression level. The determinants involved in MDR are being identified as *smeC* and *smeR*, whose deletion leads to the reversal of the resistance phenotype [172].

A similar quiescent behaviour is provided by the *smeU1VWU2X* operon, whose encoded SmeVWX proteins show 51%, 56%, and 48% amino acid identity with *P. aeruginosa* MexEF-OprN, respectively. The SmeRv protein, a LysR-type regulator, negatively regulates the operon in the *S. maltophilia* KJ strain, but it acts as a positive regulator in the *S. maltophilia* MDR KJ09C strain [170]. No mutations have been identified in *smeRv*, so the presence of an activator ligand could be able to switch on the expression of the entire operon. Differently from the other RND-efflux pumps, it possesses two additional sensor proteins, SmeU1 and SmeU2, belonging to the Short-chain Dehydrogenase/Reductase (SDR) family. The latter has been shown to mediate alleviation from environmental oxidative stress, which is found to trigger the expression of *smeU1VWU2X* [170,174]. KJ09C mutant overexpressing this operon shows increased resistance to chloramphenicol, quinolones, and tetracycline, with the MICs of aminoglycosides unexpectedly decreased [170]. Interestingly, the *smeX* deletion of KJ09C mutant reverts both the resistance and the susceptibility patterns, while *smeU2* deletion in the same strain leads only to a slight decrease in the resistance, up to a 2-fold MIC decrease in the case of aminoglycosides, suggesting an additional control exerted by SmeU2 on SmeX overexpression [174].

SmeDEF intrinsically confers a two- to eight-fold increase in the MICs of quinolones, tetracycline, chloramphenicol, and novobiocin [175]. Its components show several homologies with different Gram-negative bacterial efflux pumps: SmeD and SmeE share the highest similarities to *E. coli* AcrA and AcrE (48%) and AcrB and AcrF (61% and 58%), while SmeF is similar to SmeC (42%) [168]. The *smeDEF* operon is directly regulated by the SmeT protein, which acts as a negative regulator [176]. Different mutations in *smeT* have been linked to the acquisition of the resistance phenotype, such as L166Q and T197P, allowing tigecycline, aztreonam, and quinolones tolerance, but also fosfomycin susceptibility [169]. This pattern is reasonable, as overexpression of *smeD* and *ameF* has been linked to levofloxacin, moxifloxacin, ceftazidime, and tetracycline resistance and amikacin resistance, respectively; in addition, deletion of the *smeF* gene in K1385 and K1439 MDR strains leads to the reversion of the MDR phenotype [175]. Indirectly, the expression of this efflux pump is influenced by the SmeRySy two-component regulatory system, the main regulator of the *smeYZ* operon [176]. The deletion of these particular sensor proteins is linked with *smeDEF* up-regulation and to subsequent chloramphenicol, ciprofloxacin, tetracycline, and macrolide resistance. Counterintuitively, such deletion also increases also *smeT* expression: a possible explanation involves the presence of an intermediary modulator, whose expression is altered by *smeRySy* deletion and which mediates the interaction between SmeT and its operator, resulting in the derepression of both *smeDEF* and *smeT* [176]. Interestingly, a biocide called triclosan acts as a SmeT inactivator, consequently leading to *smeDEF* overexpression and MDR strain selection [177,178].

Two highly expressed efflux pumps, SmeYZ, and SmeIJK, play a major role in the intrinsic resistance to antimicrobials [171,173]. The *smeYZ* operon, sharing 44% and 59% amino acid identity with *Acinetobacter baumanii* AdeAB [41], confers resistance to amikacin, gentamicin, kanamycin, and leucomycin. Parallelly, its deletion leads to both aminoglycosides and trimethoprim-sulfamethoxazole susceptibility [173]. As previously stated, the operon is controlled by SmeRySy, with *smeRy* deletion downregulating *smeZ* expression and conferring aminoglycoside susceptibility, in addition to the acquired resistances involving *smeDEF* pump expression [176]. Celastrol, an anti-inflammatory natural terpenoid compound, can down-regulate *smeYZ* expression, thus proposing a possible candidate to control virulence in *S. maltophilia* [179].

*smeIJK* has a particular genetic organization, as it is the only efflux pump in *S. maltophilia* coding for two inner membrane proteins, SmeJ and SmeK, both showing high similarity (59%) among them [171]. The smeIJK operon shares 41%, 50%, and 44% amino acid identity, respectively, to MtdABC of *E. coli* [41]. SmeIJK confers intrinsic resistance to tetracycline and, to a lesser extent, to aminoglycosides; overexpression can be found in *S. maltophilia* KJ and KM5 strains leads to an up to 16-fold increase in aminoglycosides MICs and to an increase in fluoroquinolones and tetracyclines resistance, phenotypes reverted after *smeJK* deletion [167,171]. In addition, deletion of the entire operon in the KJ mutant is linked to polymyxin E susceptibility, thus suggesting a role for SmeI in membrane integrity and permeability [171].

SmeOP proteins are not conserved in other Gram-negative bacteria, as they share less than 30% of the amino acid identity of other antimicrobial efflux pumps [41]. In the strain KJ, this efflux pump is involved in the extrusion of nalidixic acid, doxycycline, macrolides, and more relevantly aminoglycosides, and in the elimination of some toxic compounds such as carbonyl cyanide 3-chlorophenylhydrazone (CCCP) and tetrachlorosalicylanilide (TCS) [180]. The operon is controlled by a TetR-type transcription regulator SmeRo, which represses the expression of the genes [180]. Its deletion only produces a slight increase in the MICs of chloramphenicol, quinolones, and tetracyclines. To properly work, the pump requires the cognate OMP TolCSm: deletion of the corresponding gene has been associated with higher decreases in the MICs than those caused by *smeOP* inactivation, suggesting the involvement of this OMP in the function of another uncharacterized efflux system [180].

Finally, *smeGH* is the last operon characterized, whose components share 39% and 49% amino acid identity to *Morganella morganii* AcrAB [41,181]. It is controlled by a TetR-type regulator, which acts as a repressor. In the *S. maltophilia* D457 strain, a *smeH* deletion mutant shows an increased susceptibility to ceftazidime, β-lactams, quinolones, and fluoroquinolones, and polymyxin B, suggesting the role of this pump in intrinsic resistance. In the same mutant, a wide variety of other noxious compounds are identified as substrates, such as menadione, benzalkonium chloride and naringenin [181]. Overexpression of *smeH* in the *S. maltophilia* clinical strain L1301 is linked to quinolones, macrolides, chloramphenicol, and tetracycline resistance; a similar effect is observed in another strain, named C2206, except for macrolide MIC, which remains unchanged [182]. Through an approach of laboratory experimental evolution, where the D457 strain was exposed to increased ceftazidime concentrations, two subsequent mutations in *smeH* were identified as linked to MDR [181]: the first to be acquired was P326Q, which confers a 5-fold and 2-fold increase in the MICs of ceftazidime and cefazolin and for aztreonam, respectively; the second acquired mutation was Q663R, which further increased the resistance to ceftazidime, cefazolin, and aztreonam, and conferred a 2-fold and 3-fold increase in the MICs of cefotaxime and norfloxacin, respectively. Finally, the role of Q663R mutation alone was explored, resulting only in a 4-fold increase in MIC of tetracycline: this suggests how relevant the order of mutation acquisition for the final phenotypic outcome is [181].

## 7. Conclusions

Multidrug-resistant strains represent a major threat for cystic fibrosis patients, who undergo heavy antibiotic therapies to face the recurrent bacterial infections that damage their lungs especially. 

Major contributors to the MDR phenotype are the efflux pumps belonging to the Resistance-Nodulation-cell Division family. These transporters are able to translocate a lot of unrelated compounds out of the bacterial cell, thus impairing the effect of the antibiotic therapy, even when a new molecule is administered for the first time [134]. Although nine families of RND proteins have been described, the Hydrophobe/Amphiphile Efflux 1 (HAE-1) is the most represented among CF bacteria, mainly being involved in the extrusion of drugs.

The contribution of this RND family in MDR has been particularly highlighted in *P. aeruginosa*, *B. cenocepacia*, *A. xylosoxidans*, and *S. maltophilia*.

In *P. aeruginosa*, six RND systems have been demonstrated to be related to the insurgence of drug resistance in clinical isolates. These pumps are involved in the extrusion of drugs belonging to different categories and were all used for the treatment of CF infections (beta-lactams, tetracyclines, fluoroquinolones, aminoglycosides, etc., Supplementary Table S1), but also detergents, dyes, and quorum-sensing signal molecules. Whole-genome sequencing of *P. aeruginosa* clinical isolates derived from CF patients revealed that, among the gene-encoding efflux pumps or their regulators, MexZ presents a high rate of mutation [183]). Indeed, a study by Henrichfreise and collaborators [184] reported that the 82% of multidrug-resistant *P. aeruginosa* strains overproduced MexXY-OprM. However, another work revealed mutations also in the efflux regulator genes *mexR*, *mexT*, and *nfxB* [185]. Non-synonymous mutations have been reported also in the transcriptional regulator of MexAB-OprM, *nalC* [186]. The same clinical isolates have mutations which lead to the activation of MexT, the positive regulator of MexEF-OprN [186]. A high mutation rate was identified also in the genes encoding the components of RND efflux pumps, such as *mexA*, *mexY*, *oprM* [187] and *mexB* [188].

In *B. cenocepacia*, sixteen genes encoding RND pumps have been identified, although a differential contribution to drug resistance has been reported when bacterial cells grow as planktonic or sessile ones [129]. Also in this case, their major role has been described for unrelated compounds, such as antimicrobial compounds, essential oils, disinfectants, and new molecules [20,130,131,132,189]. A study aimed at dissecting the mechanisms responsible for antibiotic resistance in clinical *B. cepacia* complex isolates revealed that the majority of them exhibited efflux pump activity, which correlated with resistance to various antimicrobial agents, including those used for the treatment of infections in CF patients (e.g., meropenem, ceftazidime, trimethoprim/sulfamethoxazole, Supplementary Table S1) [135]. In particular, RND-3 and RND-9 overexpression was observed in all clinical isolates, with RND-3 being the most up-regulated among the RND pumps tested [135].

During chronic infections, the long-term colonization of the lungs of CF patients is accompanied by an adaptive remodeling of the *B. cenocepacia* transcriptome. Adaptive changes include the overexpression of various genes encoding drug efflux pumps, like RND-6 and RND-4. As a consequence, the higher active drug export capacity of clinical isolates from the lungs of CF patients affected by long-term chronic infections is accompanied by an increased resistance to clinically relevant antibiotics with very different biological targets [136].

In *A. xylosoxidans*, seventeen predicted efflux systems have been reported [32]. Only three of these efflux systems have been fully characterized so far, showing the ability to confer resistance to CF used drugs, such as fluoroquinolones andtrimethoprim/sulfamethoxazole (Supplementary Table S1). As an example, Gabrielaite and collaborators [160], performing a genomic analysis on 101 clinical strains isolated within a time span of 20 years in a Denmark CF center, found that in 38% of the analyzed lineages mutations in the gene *axyZ* (*axyXY*-*oprZ* transcriptional repressor) were present. The presence of *axyZ* mutations led to an overall increase of tolerance to antibiotics since AxyXY-OprZ has a broad spectrum of activity.

Finally, in the *S. maltophilia* K279a genome eight pumps belonging to the HAE family have been annotated [168]. Their involvement in the resistance has been assessed in 102 clinical isolates, where 70%, 77%, 59% and 61% overexpressed s*meB, smeC, smeD, and smeF*, respectively [190]. In particular, as regarding the drugs currently used to treat *S. maltophilia* infections in CF (Supplementary Table S1), *smeD* overexpression was responsible for levofloxacin and minocycline resistance, *smeC* for ceftazidime and ticarcillin-clavulonate-nonsusceptibility, while *smeF* overexpression was significantly correlated with ceftazidime and levofloxacin resistance [190].

Another interesting point is that all the described pathogens are able to chronically colonize the CF airway. This implies their ability to adapt to the host environment, characterized by peculiar nutrient and oxygen availabilities, to interact with the host immune response and to deal with the presence of drugs administered to try to clear the infections. In order to understand this phenomenon, different papers reported results achieved through transcriptomics, which analyzed differential gene expression of strains isolated from CF patients, or genomic analyses which evaluated the presence of mutations in clinical isolates. Interestingly, efflux pump encoding genes were listed among those in which altered level of expression or mutations were reported as contributors to CF lung adaptation in *P. aeruginosa* [191], *B. cenocepacia* [136], *Achromobacter* sp. [146] and *S. maltophilia* [16]. This has been mainly ascribed both to their role in biofilm formation and in bacterial virulence [61], highlighting a wider role of efflux systems. Indeed, the role of RND efflux pumps in drug resistance can be demonstrated in vitro, where the amount of antibiotic can be measured, while in the clinical environment it is much more complicated to evaluate the achieved antibiotic concentrations and the consequent contribution of efflux to MDR, which might allow the acquisition of other resistance mechanisms.

Despite the recognized role in drug resistance of RND efflux transporters, more efforts are necessary to find efflux inhibitors to be administered to patients. As some molecules were shown to be effective against *P. aeruginosa* and *A. xylosoxidans*, the high degree of similarity found among the RND systems of all the described CF colonizing bacteria could lead to the discovery of new inhibitors effective against a broad range of pathogens. These molecules could be used in combination with antibiotics to avoid extrusion and MDR insurgence. Indeed, given the important contribution of specific efflux systems in the insurgence of MDR, the combined use of antibiotics and specific efflux inhibitors could represent a promising therapeutic strategy for CF patients. Interestingly, phage therapy has been shown to target specific efflux pumps in *P. aeruginosa*: this also represents a new route in the fight against drug resistance.

## Figures and Tables

**Table 1:** RND efflux pumps in *P. aeruginosa*.

RND Efflux Pump	Systematic ID	Family	Identified Regulator(s)	Substrates
MexAB-OprM	PA0425-PA0427	HAE-1	MexR, repressor (MarR-type regulator)	β-Lactams (except imipenem), β-lactam inhibitors, fluoroquinolones, tetracycline, chloramphenicol, novobiocin, macrolides, trimethoprim, triclosan (irgasan), ethidium bromide, SDS, aromatic hydrocarbons, thiolactomycin, cerulenin, acylated homoserine lactones
MexCD-OprJ	PA4599- PA4597	HAE-1	NfxB, repressor (TetR/AcrR-type regulator)	β-Lactams, fluoroquinolones, chloramphenicol, tetracycline, novobiocin, trimethoprim, macrolides, crystal violet, ethidium bromide, acriflavine, SDS, aromatic hydrocarbons, triclosan
MexEF-OprN	PA2493-PA2495	HAE-1	MexT, activator (LysR-type regulator)	Fluoroquinolones, chloramphenicol, trimethoprim, aromatic hydrocarbons, triclosan, *Pseudomonas* quinolone signal
MexXY	PA2019-PA2018	HAE-1	MexZ, repressor (TetR-type regulator	Fluoroquinolones, aminoglycosides, tetracycline, erythromycin
MexJK	PA3677-PA3676	HAE-1	MexL, repressor (TetR/AcrR-type regulator)	Tetracycline, erythromycin, triclosan
MexVW	PA4374-PA4375	HAE-1	N.D.	Norfloxacin, ofloxacin, chloramphenicol, cefpirome, tetracycline, ethidium bromide and acriflavine

**Table 2:** RND efflux pumps in *B. cenocepacia*.

RND-Efflux Pump	Systematic ID	Family	Identified Regulator(s)	Antibiotic Substrates
RND-1	BCAS0591-BCAS0593	HAE-RND	N.A.	EO
RND-2	BCAS0766- BCAS0764	HAE-RND	LysR family transcriptional regulator (BCAS0767) AraC family transcriptional regulator (BCAS0768)	Fluoroquinolones, tetracycline, rifampicin, novobiocin, EO
RND-3	BCAL1674-BCAL1676	HAE-RND	Tet-R type regulator(BCAL1672)	Nalidixic acid, ciprofloxacin, tobramycin, meropenem, chlorhexidine
RND-4	BCAL2820-BCAL2822	HAE-RND	Tet-R type regulator(BCAL2823)	Aztreonam, chloramphenicol, fluoroquinolones, tobramycin, tetracycline, rifampicin, novobiocin, essential oils, ethidium bromide, 2-thiocyanatopyridine derivative (11026103)
RND-6-7	BCAL1079-BCAL1081	HAE-RND	N.A.	EO
RND-8	BCAM0925-BCAM0927	HAE-RND	N.A.	Tobramycin
RND-9	BCAM1945-BCAM1947	HAE-RND	Mer-R type regulator(BCAM1948)	Tobramycin, chlorhexidine, EO, 2-thiocyanatopyridine derivative (11026103), 2,1,3-benzothiadiazol-5-yl family compound (10126109)
RND-10	BCAM2549-BCAM2551	HAE-RND	Tet-R type regulator (BCAM2548)	Chloramphenicol, fluoroquinolones, Trimethoprim, EO
RND-11	BCAM0711-BCAM0713	HME-RND	N.A.	Divalent cations (Zn^2+^, Co^2+^, Cd^2+^ and Ni^2+^)
RND-12	BCAM0433-BCAM0435	HME-RND	N.A.	Monovalent cations (Cu^+^ and Ag^+^), EO
RND-16	BCAL2134-BCAL2136	U.F.-RND	N.A.	Minocycline, meropenem ciprofloxacin

HAE: Hydrophobe/Amphiphile Efflux-1; HME = Heavy-Metal Efflux; U.F. = Uncertain Function. N.A. Not available; EO: Essential oils.

**Table 3:** Characterized RND efflux pumps in *A. xylosoxidans*.

RND Efflux-Pump	PAO1 Orthologous (% of Identity)	Identified Regulator(s)	Antibiotic Substrates
AxyABM	MexAB-OprM (60-72-60%)	AxyR (putative LysR-type regulator)	Cephalosporins, aztreonam, nalidixic acid, fluoroquinolones, chloramphenicol, trimethoprim/sulfamethoxazole
AxyXY-OprZ	MexXY-OprM (62-74-48%)	AxyZ (TetR-type regulator)	Aminoglycosides, carbapenems, cefepime, ceftazidime, fluoroquinolones, tetracyclines, erythromycin
AxyEF-OprN	MexEF-OprN (50-65-31%)	AxyT (LysR-type regulator)	Fluoroquinolones, carbapenems, tetracyclines

**Table 4:** RND efflux pumps in *S. maltophilia*.

RND-Efflux Pump	Systematic ID		Family	Identified Regulator(s)	Antibiotic Substrates
SmeABC	Smlt4474-4476		HAE-RND	Two-component regulator SmeSR	trimethoprim; third-generation β-lactams; aminoglycosides; fluoroquinolones
SmeDEF	Smlt4070-4072		HAE-RND	Tet-R type regulator SmeT; Two component regulator SmeRySy	chloramphenicol; ceftazidime; amikacin; aztreonam; novobiocin; fosfomycin; quinolones
SmeGH	Smlt3170-3171		HAE-RND	Tet-R type regulator	ceftazidime; tetracycline; polymyxin B; β-lactams; quinolones; fluoroquinolones
SmeIJK	Smlt4279/4281		HAE-RND	N.D.	tetracyclines; fluoroquinolones; aminoglycosides
SmeMN	Smlt3788-3787		HAE-RND	N.D.	N.D.
SmeOP	Smlt3925-3924		HAE-RND	Tet-R type regulator SmeRo	nalidixic acid; doxocycline; aminoglycosides; macrolides
SmeYZ	Smlt2201-2202		HAE-RND	Two-component regulator SmeRySy	trimethoprim-sulfamethoxazole; leucomycin; aminoglycosides
SmeU_1_VWU_2_Z	Smlt1829-1833		HAE-RND	Lys-R type regulator SmeRv	chloramphenicol; tetracycline; quinolones

N.D. Not Determined.

## Data Availability

Not applicable.

## Supplementary Materials

The following are available online at https://www.mdpi.com/article/10.3390/antibiotics10070863/s1, Table S1: Antibitoiotics used for the treatment of *P. aeruginosa*, *B. cenocepacia*, *A. xylosoxidans* ans *S. maltophilia* infections in CF patients and efflux pumps involved in resistance.
